# Multiple myeloma associated with secondary plasma cell leukemia, gastric mucosal extramedullary plasmacytic infiltration, and concurrent moderately differentiated papillary adenocarcinoma: a case report

**DOI:** 10.3389/fonc.2026.1793707

**Published:** 2026-03-19

**Authors:** Lulu Li, Wei Zheng, Minmin Yu, Jie Xu, Shumei Bai, Yueli Liu, Yan Wang, Siyuan Cui

**Affiliations:** 1 The First Clinical Medical College, Shandong University of Traditional Chinese Medicine, Jinan, China; 2 Affiliated Hospital of Shandong University of Traditional Chinese Medicine, Jinan, China

**Keywords:** case report, extramedullary disease, gastric adenocarcinoma, multiple myeloma, secondary plasma cell leukemia

## Abstract

We report a case of IgG λ–type multiple myeloma (MM) in a patient who received multiple lines of therapy and achieved repeated remissions. In 2025, the patient experienced a third relapse and had been exposed to proteasome inhibitors (PIs), immunomodulatory drugs (IMiDs), and anti-CD38 monoclonal antibodies (mAbs), meeting the criteria for triple-refractory disease. At relapse, the patient presented with fatigue and progressive anemia, and peripheral blood smear examination revealed approximately 14% atypical plasma cells, fulfilling the diagnostic criteria for secondary plasma cell leukemia (sPCL). Treatment with the KXD regimen (carfilzomib, cyclophosphamide, dexamethasone) resulted in transient disease stabilization for approximately two months, followed by further progression. During the disease course, the patient developed upper abdominal discomfort. Gastroscopy revealed a focal lesion at the gastric angle, and endoscopic submucosal dissection (ESD) was subsequently performed. Histopathological examination demonstrated a moderately differentiated intramucosal papillary adenocarcinoma of the gastric angle, with tumor infiltration confined to the mucosal layer and negative resection margins. In addition, infiltration of CD138-positive plasma cell–like tumor cells with λ light chain restriction was observed in the gastric lamina propria, consistent with extramedullary disease (EMD). The final diagnosis was relapsed MM complicated by sPCL, gastric mucosal extramedullary plasmacytic infiltration, and synchronous moderately differentiated intramucosal papillary adenocarcinoma.

## 1 Introduction

Multiple myeloma (MM) is a clonal malignant plasma cell disorder characterized by marked clinical and biological heterogeneity (1). In recent years, the introduction of proteasome inhibitors (PIs), immunomodulatory drugs (IMiDs), and monoclonal antibodies (mAbs) has significantly improved survival outcomes in patients with MM (2). However, despite continuous therapeutic advances, drug resistance, disease relapse, clonal evolution, and extramedullary progression remain formidable clinical challenges, with certain atypical disease manifestations being exceedingly rare and associated with a dismal prognosis.

Plasma cell leukemia (PCL) is a rare and highly aggressive plasma cell malignancy that may present as primary PCL (pPCL) at initial diagnosis or evolve as secondary PCL (sPCL) during the relapsed or refractory phase of MM, characterized by the presence of abundant clonal plasma cells in the peripheral blood (3). In 2021, the International Myeloma Working Group (IMWG) updated the diagnostic criteria for PCL, lowering the threshold from ≥20% to ≥5% circulating plasma cells to enhance early recognition and facilitate timely intervention (4). The prognosis of sPCL remains extremely poor, with a median overall survival of approximately 3.2 months even in the modern treatment era, substantially inferior to that of pPCL (3). In addition to hematogenous dissemination, MM may also manifest as extramedullary infiltration, termed extramedullary disease (EMD), involving soft tissues or organs outside the bone marrow, with an incidence ranging from 0.5%–5.2% at diagnosis and increasing to 5%–30% at relapse (5). Extramedullary involvement in MM is frequently associated with enhanced biological aggressiveness, with common sites including the liver, lymph nodes, soft tissues, and central nervous system, while gastrointestinal involvement is particularly rare (6). The concomitant occurrence of sPCL, gastric EMD, and synchronous primary gastric adenocarcinoma is exceedingly uncommon and poses unique challenges for clinical diagnosis and therapeutic decision-making.

This report describes a patient previously diagnosed with IgG λ–type MM who achieved long-term remission but subsequently progressed to sPCL after relapse, followed by further disease progression after a transient response. During the remission period, the patient developed gastrointestinal discomfort and underwent gastroscopic evaluation, which revealed a moderately differentiated intramucosal papillary adenocarcinoma of the gastric angle. Histopathological examination demonstrated focal infiltration of CD138-positive plasma cells with λ light chain restriction, indicating gastric mucosal EMD. This case represents a rare coexistence of sPCL, EMD, and synchronous early-stage gastric cancer. Through this case, we aim to explore the clinical characteristics and management considerations of rare late-stage disease evolution in high-risk MM and to review the relevant literature to enhance awareness of such complex clinical scenarios.

## 2 Case report

An 84-year-old woman was admitted in January 2018 for evaluation of generalized fatigue. Laboratory investigations revealed hypoalbuminemia (albumin 25.2 g/L), hyperglobulinemia (globulin 105.6 g/L), positive urinary protein, and anemia with a hemoglobin level of 81 g/L. Lactate dehydrogenase (LDH) was 88 U/L, and β2-microglobulin was 2.1 mg/L. Immunofixation electrophoresis demonstrated an IgG λ–type monoclonal protein. Quantitative immunoglobulin analysis showed IgG 91.8 g/L, IgA <0.25 g/L, and IgM 0.186 g/L. Bone marrow examination revealed plasma cells accounting for 34.5% of nucleated cells, with atypical morphology characterized by prominent nucleoli and vesicular chromatin. Flow cytometric immunophenotyping identified clonal plasma cells comprising 27.1% of nucleated cells, strongly expressing CD38 and CD138 with λ light chain restriction, negative for CD19, and partially positive for CD56. Fluorescence *in situ* hybridization (FISH) analysis demonstrated TP53 locus signal abnormality and IGH/FGFR3 fusion, while IGH/MAF fusion was negative. Based on the clinical presentation and laboratory findings, the patient was diagnosed with IgG λ–type MM, classified as ISS stage II and R-ISS stage III. The patient received four cycles of VTD (bortezomib, thalidomide, and dexamethasone) induction therapy and achieved partial response (PR). Given her advanced age, autologous stem cell transplantation was not pursued, and thalidomide was administered as maintenance therapy. In 2019, the patient experienced the first relapse and was treated with six cycles of DRD (daratumumab, lenalidomide, and dexamethasone), achieving very good partial response (VGPR), followed by maintenance therapy with daratumumab combined with lenalidomide for approximately two years. In 2021, a second relapse occurred, and the patient received eight cycles of KXD (carfilzomib, cyclophosphamide, and dexamethasone), resulting in disease stabilization with VGPR.

**Figure 1:**
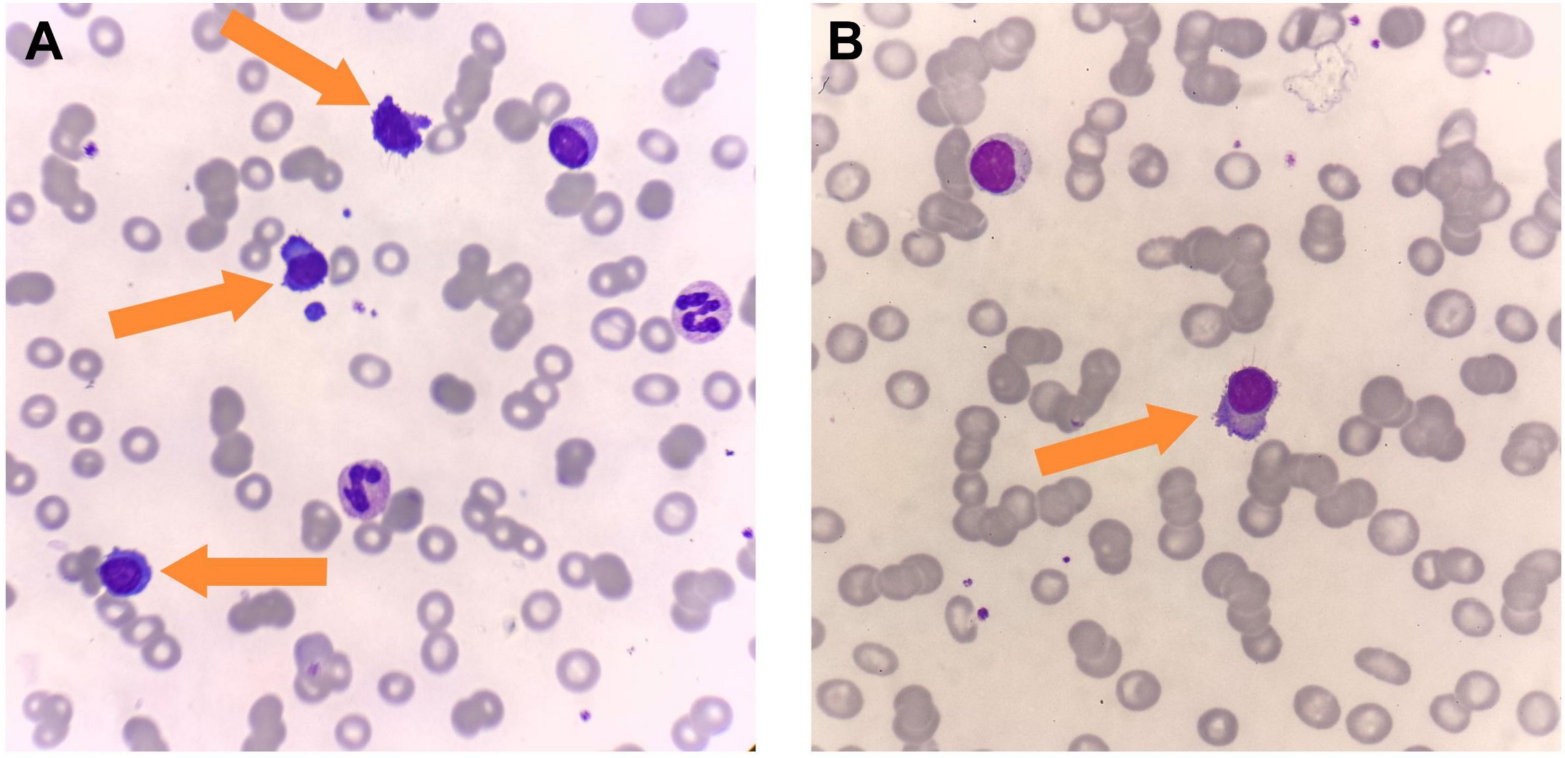
Comparison of plasma cells in peripheral blood smears. **(A)** (September 2025): Peripheral blood smear shows scattered individual plasma cells (orange arrows) with rouleaux formation of red blood cells. **(B)** (December 2025): Peripheral blood smear shows clustered plasma cells (orange arrows) with a marked increase in number compared to September but no significant morphological changes, and rouleaux formation of red blood cells.

**Figure 2:**
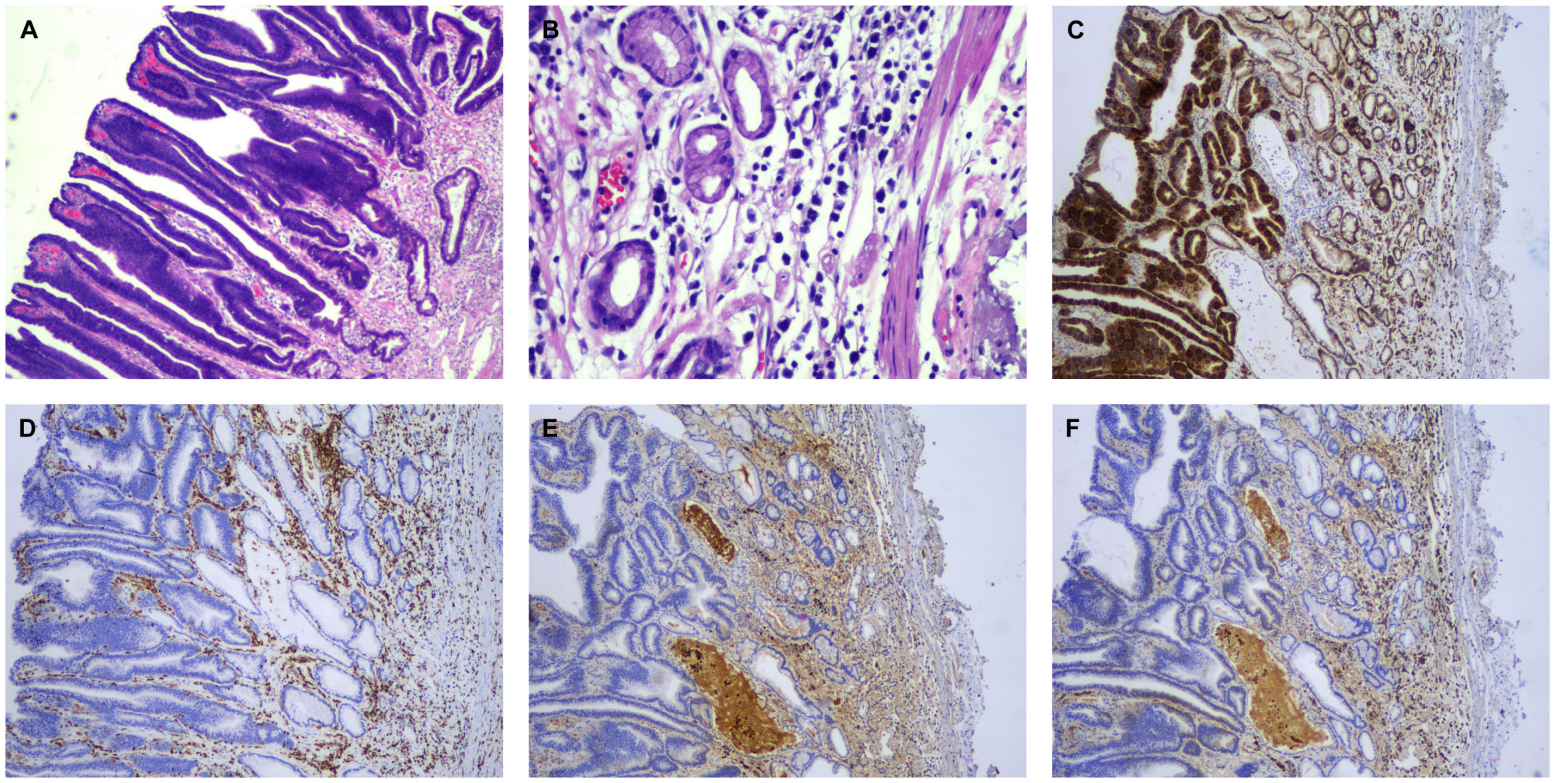
Histopathological and immunohistochemical findings of gastric specimens. **(A)** Papillary adenocarcinoma area (hematoxylin and eosin [H&E] staining, ×40). The glandular epithelium shows a papillary architecture with loss of cellular polarity, and the papillary cores consist of fibrovascular stroma. **(B)** Plasmacytoma area with tumor cell infiltration (H&E staining ×200). Tumor cells are diffusely distributed, exhibiting eccentrically located nuclei resembling immature plasma cells. An increased nuclear-to-cytoplasmic ration, hyperchromatic nuclei, eosinophilic cytoplasm, and occasional perinuclear clearing. **(C)** CD138 immunohistochemical staining (×100; DAB chromogen). Tumor cells show positive brown staining. **(D)** CD45 immunohistochemical staining (×100; DAB). Tumor cells are negative for CD45 expression. **(E)** κ light chain immunohistochemical staining (×100; DAB). Tumor cells show no κ light chain expression. **(F)** Λ light chain immunohistochemical staining (×100; DAB). Tumor cells demonstrate Λ light chain expression, indicating κ/Λ light chain restriction.

In 2025, the patient developed a third relapse. At this time, she had been exposed to PIs, IMiDs, and anti-CD38 mAbs, meeting the criteria for triple-exposed and triple-refractory disease. At the time of relapse, laboratory examination revealed a hemoglobin level of 76 g/L, and peripheral blood smear showed approximately 14% atypical plasma cells (**Figure 1A**). By December 2025, the disease progressed further, with peripheral blood smear demonstrating 19% abnormal plasma cells. Compared with the previous assessment, the number of plasma cells had increased significantly, exhibiting a scattered distribution without notable morphological changes (**Figure 1B**). In the context of her disease history, the findings were consistent with sPCL. Concurrently, the patient developed upper abdominal discomfort and underwent gastroscopic evaluation. Gastroscopy revealed a superficial elevated lesion measuring approximately 2.5 × 2.5 cm at the gastric angle, with clear borders, slightly coarse mucosa, and no obvious ulceration or bleeding. Esophagoscopy showed normal esophageal peristalsis, smooth mucosa, and no abnormalities. Subsequently, endoscopic submucosal dissection (ESD) was performed to achieve complete resection. Postoperative macroscopic pathological description: a piece of mucosal tissue from the gastric angle measuring 4.3 × 3.5 cm in area and 0.1 cm in thickness, with a superficial elevated area measuring 2.5 × 2.5 cm located 0.4 cm from the nearest edge. Microscopic examination revealed a moderately differentiated papillary adenocarcinoma of the gastric angle, infiltrating the lamina propria, with negative resection margins and no lymphovascular invasion (pT1aNxM0, stage I gastric cancer). In other areas of the gastric lamina propria, diffuse infiltration of plasmacytoid atypical cells was observed, morphologically similar to the neoplastic plasma cells in the bone marrow. Immunohistochemistry showed positivity for CD138, λ light chain restriction, and a Ki-67 proliferation index of approximately 80%. Based on the morphological and immunophenotypic findings, MM-related gastric mucosal EMD was diagnosed (**Figure 2**). The final diagnosis was relapsed IgG λ–type MM (high-risk R-ISS, triple-refractory), complicated by sPCL, gastric mucosal EMD, and synchronous moderately differentiated intramucosal papillary adenocarcinoma. The gastric adenocarcinoma was treated successfully with ESD, achieving negative margins and meeting the criteria for curative resection. For relapsed MM complicated by sPCL and gastric EMD, the patient subsequently received teclistamab therapy. During the treatment course, the patient developed grade 2 cytokine release syndrome (CRS), which was rapidly controlled. After one treatment cycle, the patient achieved partial response (PR) again and is currently continuing teclistamab therapy.

## 3 Discussion

The clinical course of this patient was exceptionally complex, reflecting the heterogeneity and aggressive nature of advanced-stage MM. Initially diagnosed with IgG λ–type MM, the patient experienced multiple cycles of remission and relapse. At the third relapse in 2025, the disease not only evolved into sPCL but was also accompanied by the rare occurrence of gastric mucosal EMD. During the same period, a moderately differentiated intramucosal papillary adenocarcinoma of the stomach was diagnosed. The exceedingly rare coexistence of these three entities suggests that in advanced MM, the disease may transcend traditional bone marrow-dependent biological behavior, presenting a complex clinical picture characterized by systemic dissemination and the synchronous occurrence of multiple primary tumors.

EMD represents an aggressive disease manifestation of MM and is generally associated with an adverse prognosis. Gastrointestinal involvement is relatively uncommon and has mostly been reported in isolated case reports. A large retrospective study demonstrated that among 2,584 patients with MM, biopsy-proven gastrointestinal involvement accounted for only 0.9% of cases, with the majority occurring during disease relapse (7). The clinical presentation of gastric involvement is highly nonspecific, ranging from mild epigastric discomfort, nausea, and vomiting—which can easily be mistaken for chemotherapy-related adverse effects or benign gastric disorders—to acute emergencies such as life-threatening upper gastrointestinal bleeding caused by tumor erosion, necessitating palliative total gastrectomy (8), or biliary or pancreatic duct obstruction presenting with jaundice and abdominal pain that may be radiologically indistinguishable from pancreatic carcinoma (9). Additionally, sporadic cases have described the coexistence or sequential occurrence of gastric plasmacytoma and primary gastric adenocarcinoma, underscoring the need for heightened vigilance for multiple lesions during the diagnostic process (10). In the present case, the patient presented with epigastric discomfort, and gastroscopy with biopsy confirmed early gastric adenocarcinoma at the gastric angle accompanied by plasmacytic infiltration, highlighting that when MM patients develop new or persistent gastrointestinal symptoms, timely endoscopic and pathological evaluation should be pursued. ^18^F-FDG PET/CT offers advantages in detecting metabolically active extramedullary lesions (11). However, PET-CT was not performed in this case, which represents a limitation of this study. Definitive diagnosis still relies on histopathological and immunohistochemical analysis of endoscopic biopsy specimens, typically demonstrating CD138 positivity with light chain restriction, and requires differentiation from lymphoma and poorly differentiated carcinoma.

In this case, gastric plasmacytic infiltration coexisted with primary gastric adenocarcinoma, representing an extremely rare example of dual malignancies. The clinical significance lies in the necessity to distinguish lesions of different biological origins, which poses diagnostic and therapeutic challenges. Although no direct evidence currently supports a causal relationship between the two tumors, chronic inflammation and immune dysregulation are considered important background factors contributing to the development of second primary malignancies in patients with MM (12). Previous case reports have described the coexistence of MM and gastric adenocarcinoma, with authors suggesting that the development of these tumors may be associated with defective immunosurveillance and chronic inflammation related to autoimmune diseases (13). Alternatively, pre-existing gastric mucosal pathology may lead to alterations in the local mucosal barrier and immune microenvironment, providing a permissive niche for the localized expansion of plasma cell clones (14). This reminds us that comprehensive evaluation should be maintained in patients with a history of MM during clinical diagnosis and treatment to avoid overlooking the presence of second tumors. However, in the present case, neither tumor microenvironment analysis nor molecular sequencing was performed, nor were comparisons made between bone marrow and gastric plasma cells regarding immunophenotypic profiles or proliferation indices. Therefore, the aforementioned mechanisms remain speculative based on previous literature and warrant further exploration in future studies.

The rapid development of sPCL and gastric mucosal extramedullary infiltration following relapse in this patient is closely associated with her high-risk genetic background. The patient harbored an IGH/FGFR3 fusion [corresponding to t(4,14)] chromosomal translocation, a well-established high-risk cytogenetic abnormality in MM. Studies have demonstrated that t(4,14) promotes upregulation of FGFR3 and MMSET expression, reducing the adhesion dependence of plasma cells on the bone marrow microenvironment and thereby enhancing their migratory capacity, facilitating extramedullary dissemination and evolution to sPCL (15). Research has also reported that the detection rate of t(4,14) translocation is significantly higher in patients with EMD and sPCL compared to the general MM population, suggesting a strong association with highly aggressive phenotypes (6). Moreover, in patients with relapse and extramedullary transformation, t(4,14) abnormalities frequently co-occur with other high-risk clonal events, exhibiting a “multi-hit” pattern that further promotes rapid disease progression (16). Therefore, in MM patients harboring such high-risk genetic abnormalities, vigilance should be maintained regarding the risk of extramedullary and leukemic transformation, with enhanced dynamic monitoring and individualized treatment management. The rapid evolution observed in this case aligns with the aforementioned high-risk biological features; however, due to the lack of longitudinal molecular monitoring, the precise clonal evolution trajectory remains to be elucidated.

This case offers several important clinical insights. In MM patients with high-risk genetic abnormalities, heightened vigilance is warranted regarding the risk of extramedullary invasive evolution. During follow-up, the emergence of new or persistent nonspecific symptoms should prompt timely imaging and pathological evaluation to avoid delayed diagnosis. Furthermore, given the disease-related immune dysregulation and the cumulative impact of long-term therapy, such patients may have an increased risk of developing second primary malignancies; therefore, when new lesions are detected, their nature requires careful differentiation. Overall management should be guided by multidisciplinary collaboration and systematic monitoring strategies to optimize individualized treatment decisions.

## Data Availability

The original contributions presented in the study are included in the article. Further inquiries can be directed to the corresponding author.

## References

[1] Malard F Neri P Bahlis NJ Terpos E Moukalled N Hungria VTM . Multiple myeloma. Nat Rev Dis Primers. (2024) 10:45. doi: 10.1038/s41572-024-00529-7, PMID: 38937492

[2] Zanwar S Rajkumar SV . Current risk stratification and staging of multiple myeloma and related clonal plasma cell disorders. Leukemia. (2025) 39:2610–7. doi: 10.1038/s41375-025-02654-y, PMID: 40702148 PMC12589131

[3] Singh S Peshin S Wertheim BC Larsen A Chineke I Sborov DW . Outcomes and treatment patterns in primary and secondary plasma cell leukemia: insights from a large us cohort study. Haematologica. (2025) 110:2129–38. doi: 10.3324/haematol.2024.287158, PMID: 40241568 PMC12399943

[4] Fernández de Larrea C Kyle R Rosiñol L Paiva B Engelhardt M Usmani S . Primary plasma cell leukemia: consensus definition by the international myeloma working group according to peripheral blood plasma cell percentage. Blood Cancer J. (2021) 11:192. doi: 10.1038/s41408-021-00587-0, PMID: 34857730 PMC8640034

[5] Ho M Paruzzo L Minehart J Nabar N Noll JH Luo T . Extramedullary multiple myeloma: challenges and opportunities. Curr Oncol (Toronto Ont). (2025) 32. doi: 10.3390/curroncol32030182, PMID: 40136386 PMC11940950

[6] Bladé J Beksac M Caers J Jurczyszyn A von Lilienfeld-Toal M Moreau P . Extramedullary disease in multiple myeloma: A systematic literature review. Blood Cancer J. (2022) 12:45. doi: 10.1038/s41408-022-00643-3, PMID: 35314675 PMC8938478

[7] Talamo G Cavallo F Zangari M Barlogie B Lee CK Pineda-Roman M . Clinical and biological features of multiple myeloma involving the gastrointestinal system. Haematologica. (2006) 91:964–7. 16818286

[8] Miyamoto K Fujii A Tada T Nakayama K Urazumi K . Total gastrectomy for gastric invasion of multiple myeloma-a case report. Jpn J Cancer Chemother (2023) 50:1120–2. 38035850

[9] Lin HH Lin Y-C Chen J-H Wang Y-C . Relapsed multiple myeloma with gastric and pancreatic extramedullary plasmacytomas. J Formosan Med Assoc. (2023) 122:355–7. doi: 10.1016/j.jfma.2023.01.001, PMID: 36693771

[10] Kusano Y Nishimura N Yokoyama M Terui Y Hatake K . Gastric involvement, a rare site for extramedullary myeloma. Ann Hematol. (2016) 95:663–4. doi: 10.1007/s00277-016-2596-z, PMID: 26754637

[11] Gülbahar Ateş S Uçmak G . A rare extramedullary involvement site in patients with multiple myeloma: stomach. Clinical Nuclear Medicine (2022) 47:e702–e3. doi: 10.1097/rlu.0000000000004299, PMID: 35619203

[12] Musto P Anderson KC Attal M Richardson PG Badros A Hou J . Second primary Malignancies in multiple myeloma: an overview and imwg consensus. Ann Oncol. (2017) 28:228–45. doi: 10.1093/annonc/mdw606, PMID: 27864218

[13] Al-Ammari M Adam S . Concomitant multiple myeloma, gastric adenocarcinoma and evan’s syndrome in a patient presenting with anaemia. BMJ Case Rep. (2016) 2016. doi: 10.1136/bcr-2016-217697, PMID: 27979847 PMC5174897

[14] Holler A Cicha I Eckstein M Haderlein M Pöttler M Rappl A . Extramedullary plasmacytoma: tumor occurrence and therapeutic concepts-a follow-up. Cancer Med. (2022) 11:4743–55. doi: 10.1002/cam4.4816, PMID: 35578404 PMC9761078

[15] McAvera R Quinn J Murphy P Glavey S . Genetic abnormalities in extramedullary multiple myeloma. Int J Mol Sci. (2023) 24. doi: 10.3390/ijms241411259, PMID: 37511018 PMC10379577

[16] Smetana J Kuglík P Grešliková H Kupská R Němec P Mikulasova A . Clonal cytogenetics changes in progression of multiple myelomato extramedullary relapse and plasmocellular leukemia: A casereport. Int J Clin Exp Pathol (2016) 9:49–60.

